# Supplementary material for: Multidrug Resistant Coagulase-Positive Staphylococcus aureus and Their Enterotoxins Detection in Traditional Cheeses Marketed in Banat Region, Romania
Source: Antibiotics (Basel). 2021 Nov 26;10(12):1458. doi: 10.3390/antibiotics10121458 (PMC8698683; doi:10.3390/antibiotics10121458)
Supplement: Supplementary file 1 [file antibiotics-10-01458-s001.zip › antibiotics-1467510-supplementary.pdf]

Supplementary Materials

Table S1. Origin, antimicrobial resistance (full circles) and susceptibility (empty circles) profile of the tested *S. aureus* strains with the AST-P592 card, and isolated in the period February 2012 and October 2013

**Antimicrobial**			**No. of tested *S. aureus* strains according to their origin**																			
**Class**	**Agent**	**MIC range µg/mL**	**telemea**								**caș**				**burduf**		**urdă**	**caș**				
			**sheep milk**															**cow milk**				
			1	2	3	4	5	6	7	8	9	10	11	12	13	14	15	16	17	18	19	20
**ß – lactams**	PCG	0.03-0.5	○	○	○	●	●	●	●	○	○	●	○	●	○	●	●	○	○	○	○	○
	OXA	0.25 – 4	○	○	○	○	○	●	●	●	○	●	○	○	○	○	○	○	○	○	○	○
	IPM	1 – 16	○	○	○	○	○	●	●	○	○	●	○	○	○	○	○	○	○	○	○	○
**aminoglycosides**	GEN	0.5 – 16	○	○	○	○	○	○	○	○	○	○	○	○	○	○	○	○	○	○	○	○
**fluoroquinolones**	CIP	0.5 – 8	○	○	○	○	●	●	○	○	●	○	●	○	○	○	○	●	○	●	○	○
	MXF	0.25 – 8	○	○	○	○	○	○	○	○	○	○	○	○	○	○	○	○	○	○	○	○
**steroids**	FA	0.5 – 32	○	○	○	○	○	○	●	●	○	●	○	●	○	○	○	○	○	○	○	○
**glycopeptides**	TEC	0.5 – 32	●	○	○	○	○	○	●	○	○	●	○	○	○	○	○	○	●	●	○	○
	VAN	0.5 – 32	○	○	○	○	○	○	●	○	○	●	○	○	○	○	○	○	○	○	○	○
**lincomycins**	CLI	0.25 – 8	○	○	○	●	○	○	●	●	●	●	○	○	○	○	○	○	○	○	○	○
**macrolides**	ERY	0.25 – 8	○	○	○	●	○	○	●	○	○	●	○	○	○	○	○	○	○	○	○	○
**oxazolidinones**	LZD	0.5 – 8	○	○	○	○	○	○	●	●	○	●	○	○	○	○	○	○	○	○	○	○
**phosphonic acid derivative**	FOF	8 – 128	○	○	○	○	○	○	○	○	○	○	○	●	○	○	○	○	○	○	○	○
**rifamycins**	RIF	0.5 – 32	●	●	●	●	●	●	●	●	●	●	○	○	○	○	○	○	○	○	○	○
**sulfonamides**	SXT	10 – 320	○	○	○	○	○	○	○	○	○	○	○	○	○	○	○	○	○	○	○	○
**tetracyclines**	TET	1 -16	○	○	○	●	○	○	●	○	○	○	●	○	○	○	○	○	○	○	○	○
	TGC	0.12 – 2	○	○	○	○	○	○	○	○	○	○	○	○	○	○	○	○	○	○	○	○
**No. of classes with resistance**			2	1	1	4	3	3	8	5	3	7	2	3		1	1	1	1	2		

Legend: MIC – minimum inhibitory concentration; PCG - benzylpenicillin; OXA – oxacillin; IPM – imipenem; GEN – gentamicin; CIP – ciprofloxacin; MXF – moxifloxacin; FA – fusidic acid; TEC – teicoplanin; VAN – vancomycin; TGC – tigecycline; CLI – clindamycin; ERY – erythromycin; LZD – linezolid; FOF – fosfomycin; RIF – rifampicin; SXT – trimethoprim – sulfamethoxazole; TET – tetracycline.

Table S2. Origin, antimicrobial resistance (full circles) and susceptibility (empty circles) profile of the tested *S. aureus* strains with the AST-GP69 card, and isolated in the period February 2012 and October 2013

**Antimicrobial**			**No. of tested *S. aureus* strains according to their origin**																	
			**caș**			**telemea**														
			**sheep milk**		**cow milk**	**sheep milk**											**cow milk**			
**Class**	**Agent**	**MIC range µg/mL**	1	2	3	4	5	6	7	8	9	10	11	12	13	14	15	16	17	18
**ß lactams**	PCG	0.03 – 0.5	○	○	○	○	○	●	●	○	○	●	●	●	○	○	●	●	○	○
	OXA	0.25 – 4	○	○	○	○	○	●	○	○	○	○	○	○	○	○	○	○	○	○
	AMP	2 – 64	○	○	○	○	○	●	○	○	○	○	○	○	○	○	○	○	○	○
	IPM	1 – 8	○	○	○	○	○	●	○	○	○	○	○	○	○	○	○	○	○	○
	SAM	2 – 64	○	○	○	○	○		○	○	○	○	○	○	○	○	○	○	○	○
**aminoglycosides**	GEN	0.5 - 16	○	○	○	○	○	○	○	○	○	○	○	○	○	○	○	○	○	○
	KAN	0.25 – 0.64	○	○	○	○	○	○	○	○	○	○	●	●	○	○	○	○	○	○
**quinolones**	ENR	0.25 – 16	●	●	●	●	●	●	●	●	●	●	●	○	●	○	●	○	●	○
	MBX	0.25 – 8	○	○	○	○	○	○	○	○	○	○	○	○	○	○	○	○	○	○
**macrolides**	ERY	0.25 – 16	○	○	○	○	○	○	○	○	○	○	○	○	○	○	○	○	○	○
**lincomycins**	CLI	0.25 – 16	○	○	○	○	○	○	○	○	○	○	○	○	○	○	○	○	○	○
**tetracyclines**	TET	2 – 32	○	○	○	○	○	●	●	○	○	●	●	●	○	○	○	●	●	●
**glycopeptides**	VAN	0.25 – 8	○	○	○	○	○	●	○	○	○	○	○	○	○	○	○	○	○	○
**steroids**	FA	1 – 16	○	○	○	○	○	●	○	○	○	○	○	○	○	○	○	○	○	○
**rifamycins**	RIF	0.5 – 8	○	○	○	○	○	●	●	●	●	●	○	○	○	○	○	○	○	○
**nitrofuran derivate**	NIT	16 – 512	○	○	○	○	○	○	○	○	○	○	○	○	○	○	○	○	○	○
**pseudomonic acid derivative**	MUP	0.06 – 512	○	○	○	○	○	●	○	○	○	○	○	○	○	○	○	○	○	○
**amphenicols**	CHL	4 – 32	○	○	○	○	○	○	○	○	○	○	○	○	○	○	○	○	○	○
**sulfonamides**	SXT	20 – 76	○	○	○	○	○	○	○	○	○	○	○	○	○	○	○	○	○	○
**No. of classes with resistance**			1	1	1	1	1	7	4	2	2	4	4	3	1		2	2	2	1

Legend: MIC – minimum inhibitory concentration; PCG - benzylpenicillin; OXA – oxacillin; AMP – ampicillin; IPM – imipenem; SAM - ampicillin/sulbactam; GEN – gentamicin; KAN – kanamycin; ENR – enrofloxacin; MBX – marbofloxacin; FA – fusidic acid; VAN – vancomycin; CLI – clindamycin; ERY – erythromycin; RIF – rifampicin; NIT – nitrofurantoin; MUP – mupirocin; CHL – chloramphenicol; SXT – trimethoprim – sulfamethoxazole; TET – tetracycline.

Table S3. Origin, antimicrobial resistance (full circles) and susceptibility (empty circles) profile of the tested *S. aureus* strains with the AST—GP79 card, isolated in the period January 2020 – February 2021

**Antimicrobial**			**No. of tested *S. aureus* strains according to their origin**										
			telemea									caș	
			sheep milk						cow milk			cow milk	
**Class**	**Agent**	**MIC range µg/mL**	1	2	3	4	5	6	7	8	9	10	11
**ß – lactams**	PCG	0.03 – 64	●	●	●	●	●	●	●	●	●	●	●
	OXA	0.25 – 4	●	○	○	○	○	●	○	○	○	●	○
	AMP	0.25 – 16	○	○	○	○	○	○	○	○	○	○	○
	CET	2 – 32	○	○	○	○	○	○	○	○	○	○	○
	CTF	0.5 – 8	○	●	●	●	●	○	●	●	●	○	●
	CEF	1 – 64	○	○	○	○	○	○	○	○	○	○	○
**aminoglycosides**	GEN	0.5 – 16	○	○	○	○	○	●	○	○	○	●	○
	KAN	4 – 64	○	●	●	●	●	●	●	●	●	●	●
	AMK	2 – 64	○	●	●	●	●	●	●	●	●	●	●
	NEO	2 – 32	○	●	○	●	○	●	●	●	●	○	●
**quinolones**	ENR	0.5 – 4	●	●	●	●	●	●	●	●	●	●	●
**macrolides**	ERY	0.25 – 8	○	●	●	●	●	○	●	●	●	○	●
	TMS	0.25 – 4	●	○	○	○	○	●	●	○	○	●	○
	TYL	1 – 32	○	○	○	○	○	●	○	○	○	●	○
**lincomycins**	CLI	0.125 – 4	○	●	●	●	●	●	●	●	●	●	●
**tetracyclines**	TET	1 – 16	○	●	●	●	●	○	●	●	●	○	●
**amphenicols**	FLO	4 – 32	○	○	○	○	○	○	○	○	○	○	○
**sulfonamides**	SXT	10 – 320	○	○	○	○	○	○	○	○	○	○	○
**No. of classes with resistance**			3	6	6	6	6	5	6	6	6	5	6

Legend: MIC – minimum inhibitory concentration; PCG - benzylpenicillin; OXA – oxacillin; AMP – ampicillin; CET – cefalotin; CTF – ceftiofur; CEF – cefquinome; GEN – gentamicin; KAN – kanamycin; AMK – amikacin; NEO – neomycin; ENR – enrofloxacin; ERY – erythromycin; TMS – tilmicosin; TYL – tylosin; CLI – clindamycin; TET – tetracycline; FLO – florfenicol; SXT – trimethoprim – sulfamethoxazole.
